# A mini review of communicative language testing

**DOI:** 10.3389/fpsyg.2023.1058411

**Published:** 2023-04-06

**Authors:** Linyu Liao, Shelly Xueting Ye, Jinsong Yang

**Affiliations:** ^1^School of Foreign Languages, Guangdong Medical University, Dongguan, China; ^2^Department of English, University of Macau, Macau, China

**Keywords:** communicative language testing, approaches to CLT, challenges of CLT, language assessment, communicative abilities

## Abstract

Compared with traditional language testing, which heavily emphasizes psychometric reliability, communicative language testing (CLT), which uses authentic tasks to measure communicative abilities, has long been dominant in language assessment. Given its widely acknowledged advantages and widespread use, CLT has become less controversial in the language assessment field and thus is receiving decreased scholarly attention. However, real-world communication, in which CLT is grounded, evolves over time, suggesting the need to update our understanding of it. To address this need and facilitate the further development of CLT theories and practices, this paper offers an up-to-date review of CLT, including its various approaches, implementation challenges, and suggestions for future research.

## Introduction

As the communicative approach to language teaching gained popularity in the 1970s, communicative language testing (CLT) appeared alongside it and has gradually taken over language assessment in recent decades. Nowadays, most large-scale tests make the marketing claim that they assess test takers' communicative language ability either explicitly or implicitly (Fulcher, 2000). Contrary to conventional tests that rely heavily on achieving reliability through multiple choice items (see Lado, 1961), CLT stresses the test takers' ability to accomplish communicative purposes appropriately within authentic contexts (Morrow, 2018). Instead of assessing language knowledge (e.g., lexical, grammatical, or phonological knowledge) that test takers possess, CLT seeks to evaluate what they can do with the language (Morrow, 2012).

Because of these distinctive features, CLT has been widely used in test development practice and is thought to have become one of the important norms of test development (Fulcher, 2000). Given its prominent role and widespread use, CLT is generally considered an uncontroversial topic with little need for in-depth investigation or discussion when compared to other topics in language assessment. As a result, the considerable research attention that was given to CLT throughout the first decade after it was proposed has since decreased (Morrow, 2012). Meanwhile, it should be noted that CLT is grounded in real-world communication, which is constantly changing as time and technology progress (McNamara, 1996). These changes have facilitated the emergence of various CLT approaches as well as new challenges (Harding, 2014). Thus, the extant CLT research and discussions, most of which were undertaken decades ago (e.g., Canale and Swain, 1980; Canale, 1983), may not accurately reflect the current state of CLT and may fail to appropriately guide the development of language tests that can assess contemporary communicative language abilities. Therefore, to ensure the sustainable use of CLT, continuous and sufficient research should be conducted, particularly reviews that can present an accurate and up-to-date state of CLT. In response to this need, this paper offers a short and concise review of CLT that includes an overview of the present CLT approaches, the as-yet-unsolved challenges to its implementation in large-scale assessments, and recommendations for future CLT research. Given the comprehensive updates provided in this review, it has significant implications for test developers and CLT researchers.

## Approaches to CLT

According to Bachman's (1990) and McNamara's (1996) introductions and Harding's (2014) summary of CLT approaches, the various CLT approaches have been classified into three categories: the theory-based approach, the real-life approach, and the integrated approach. The features of each of these are summarized below.

### The theory-based approach

This approach focuses on the measurement of underlying traits of communicative language ability (CLA). Bachman's (1990) model of CLA in use clarified that CLA includes language competences (e.g., vocabulary, syntax) and strategic competences (e.g., goal setting, planning, assessment). Accordingly, language tests developed using the theory-based approach and those specifically based on the Bachman (1990) model should contain tasks that can measure the model's stated competencies (Fulcher and Davidson, 2007). Among the illustrative projects provided by Bachman and Palmer (1996) to explain their use of this approach in test development is a test developed for a hotel seeking to hire employees to handle written English complaints. The linguistic and strategic competences necessary for this position were specified in detail and thus could be measured by the test. For instance, knowledge of English vocabulary for hotel facilities and services, as one of the language competencies required in the target domain, could be assessed by a task that asks test takers to provide the form and meaning of target words. Similarly, the strategic competency of assessment, which in this context refers to the evaluation of the appropriateness of responses to complaints, could be measured by a task requiring candidates to choose the best answers to a range of inquiries and complaints. Bachman (1990) and McNamara (1996) also referred to this approach as the interactional authenticity approach and the cognitive/psycholinguistic tradition, respectively. Given that these two approaches have the overlapping feature of measuring language skills based on theoretical models of CLA (e.g., Canale and Swain, 1980; Canale, 1983; Bachman, 1990; Bachman and Palmer, 1996, 2010; Chapelle, 1998; Douglas, 2000; Purpura, 2004), they are both regarded as falling under the category of the theory-based approach (Harding, 2014). According to these existing models, it is generally accepted that CLA is a multi-componential construct. However, consensus has not yet been reached on which components account for a comprehensive model of this construct (e.g., McNamara, 1996; Van Moere, 2012). For example, it is uncertain whether the competences of managing emotional response (Lindemann, 2002) or comprehending different English varieties (Canagarajah, 2006) should be incorporated into the CLT construct. Thus, the fundamental question of which specific components of CLA should be assessed still confronts the theory-based approach to CLT.

### The real-life approach

The real-life approach is atheoretical but authentic. It is also known as the real-life task-driven approach by Bachman (1990) and the work-sample tradition by McNamara (1996). This approach begins with the analysis of the target language use domain, which is then followed by the development of test tasks simulating real-life activities of language use. For example, language tests aimed at assessing prospective university students' English academic writing should include integrated listening-and-writing tasks that simulate the common university practice of completing a writing assignment after attending a lecture. It can be said that the real-life approach emphasizes simulation in test design (McNamara, 1996), and therefore prioritizes integrated tasks that represent language use in authentic contexts better than traditional independent tasks do. This approach has grown in popularity in recent years (see Cumming, 2014). In addition to being authentic, test tasks also need to be sufficiently diversified to elicit enough evidence of test takers' CLA. However, without practical guidance for using the real-life approach for CLT, the sampling and design of authentic and varied test tasks can be challenging due to the complexity of real-life communication.

### The integrated approach

The integrated approach is the combination of the theory-based approach and the real-life approach. On the one hand, it draws on existing theories of CLA to assess the underlying traits of communicative competence. On the other hand, it utilizes real-life tasks representative of target language use domain to ensure test authenticity. A clear example of this approach comes from a test designed to recruit administrative staff at an English-medium university based on Bachman and Palmer's (1996) illustrative projects. Since one of most frequent duties of this job is typing in handwritten documents, a test developed using the real-life approach might include tasks that require test takers to demonstrate their typing skills but not necessarily their English language competencies. However, applicants' subsequent performances on these tasks would probably fail to fully represent how well they will perform in this position. Given this, the theory-based approach, which requires the specification of linguistic and strategic competences that are necessary in the target context prior to test development, can be utilized in conjunction with the real-life approach to better serve the test purpose. This integrated approach could, for instance, develop a task requiring test takers to write English emails to announce department news, replicating the aforementioned real-life typing activities while also eliciting a demonstration of the test takers' English competencies. The integrated approach, which makes use of the strengths of both traditional approaches, is now regarded as the mainstream approach to CLT.

## Challenges of CLT

### The difficulty of construct operationalization

A long-standing CLT challenge is the difficulty of operationalizing CLA models for a specific context or test design. For example, when McNamara (1990) first utilized the innovative item response theory to validate language tests, he not only noted the general difficulty in implementing CLT in practice but also suggested that this challenge might be related to raters' varied perceptions of the importance of particular CLA components. Subsequent literature has also accepted this claim (Harding, 2014). McNamara (2003) in a later review observed that Bachman's model had seldom been used in test development projects. A similar observation was also made by Linda Taylor (as cited in Harding, 2014) while attempting to implement Bachman's (1990) CLA model in the International English Language Testing System (IELTS). She found it extremely challenging to turn CLT models into practical step-by-step instructions, which again emphasizes the difficulty in operationalizing the constructs of complex CLA frameworks. Some efforts have been made to solve this challenge. For instance, reports that describe the test development process of both the Common European Framework of Reference (CEFR) and the Educational Testing service's (ETS). Test of English as a Foreign Language (TOEFL) (e.g., Bejar et al., 2000; Butler et al., 2000) have been made public to assist test developers in better applying CLT to their assessment design. Additionally, recent research has explored people's perceptions of communicative ability from various perspectives (e.g., Plough et al., 2010; Abdul Raof, 2011; Kim and Elder, 2015; Pill, 2016), the findings of which have benefited the development of analytic assessment criteria. These criteria, which provide clearer descriptions of CLT constructs, might serve as practical references for test development based on sophisticated CLT frameworks. For example, parts of the rating criteria for evaluating oral communicative competence, which were initially identified by Davies et al. (1999), have been used by Pearson Education (2022) for its test of general English. In spite of the attempts that have been made during the past three decades, the notable challenge of construct operationalization has not yet been fully resolved and should continue to receive attention in the 21st century. As Harding (2014) comments, “[i]ncreasingly complex frameworks of communicative language ability may hinder as much as enable good test design” (p. 191) because they are too complex to be applied.

### The inadequacy of construct definitions

There is also an issue with the definitions of the constructs of CLA. On the one hand, exiting models tend to be too complex to be applied in practice and thus can only partially be validated (e.g., Harley et al., 1990; Phakiti, 2008). On the other hand, they are not yet complex or rich enough to provide a comprehensive definition of CLA (Purpura, 2008; Harding, 2014). Some alternative CLA models have been proposed to try and cover CLA components overlooked in older models. For example, Purpura (2008) suggests that existing models should be supplemented with theories of Second Language (L2) learning and development. L2 is defined as the language that people use other than their mother tongue (Lado, 1957). However, a consensus on exactly which components constitute a comprehensive CLA model has not been reached. Moreover, CLA is not a static construct. No matter how comprehensive a CLA model is, it cannot satisfy the needs of each subsequent generation without constant evolution. According to Harding (2014), the theoretical constructs of CLA should incorporate changes to communicative practices brought about by technology such as those of online and mobile communication. He also highlighted a number of abilities that were neglected in the past but have eventually come to be recognized as CLA components as technology has advanced and communicative practices changed. Typical examples include (1) the ability to interact with interlocutors in paired or group conversations (Taylor and Wigglesworth, 2009), (2) the ability to adapt to various language varieties (Leung, 2005), and the ability to employ socio-pragmatic knowledge while communicating (Timpe, 2012). Additionally, it should be noted that test developers and experts in task content have different opinions about which CLA constructs are important, with the former focusing primarily on linguistic features and the latter emphasizing non-verbal elements and behaviors that aid in learning (Sato, 2018). Given this, if the CLT constructs were specified by test developers alone, they might fail to accurately reflect some specific context's communicative needs (Wette, 2011). Therefore, it is crucial to combine both perspectives in order to elicit an adequate definition of the CLT constructs.

### Feasibility in large-scale tests

CLT requires that test tasks be as naturalistic and authentic as possible, replicating real-life language use activities. Despite that this contextualized approach is beneficial and feasible in classroom settings, it can complicate assessment by introducing a variety of task demands and contextual factors, especially for large-scale tests (Morrow, 2018). Some tasks such as presentations, although authentic and practical, are difficult to incorporate into large-scale assessment. Since tasks of this type require particular knowledge and skills, test takers may not be able to accomplish them even in their first language without purposeful training and practice. Another example of this problem is the utilization of paired and group assessments to measure test takers' interaction skills in real-world situations (Taylor and Wigglesworth, 2009). Test takers' performances on these tasks involve multiple individuals, and therefore a single performance might not accurately represent that test taker's ability since they might be disadvantaged by being unlucky enough to be assigned to less-engaged or skilled partners (Isaacs, 2013). Furthermore, the ability to understand diverse language varieties, which is becoming increasingly important in communication today, is one of the CLA constructs that some assert should be measured in language assessments (e.g., Taylor and Geranpayeh, 2011; Ockey et al., 2016). However, test developers are responding cautiously to this suggestion (Elder and Harding, 2008) given their concern over the potential issue of test bias when various varieties are added. That is, test takers tend to differ in their familiarity with language varieties, which likely affects their test performance and thus leads to unfairness (Ockey and Wagner, 2018). As a result, various compromises for feasibility must be made when designing authentic tasks for large-scale tests.

### The tension between face validity and reliability

CLT emphasizes face validity by requiring test takers to produce actual language in interactive tasks that resemble real-life communication. This, however, tends to reduce test reliability when compared with conventional discrete-point test items that focus on specific elements of language knowledge or skill because language performance involving qualitative rating is notoriously difficult to score reliably. In addition, since interactive tasks require both receptive and productive skills, tests may not be able to reliably assess test takers' real CLA if they are not equally capable in both types of skills. This is particularly true for test takers with a lower language proficiency. As Morrow (2018) explains, since beginners are usually better at comprehending language than producing it, their limited language performance cannot represent or capture the development of their receptive skills. Additionally, it is said that assessments based on CLT theories should measure not only linguistic knowledge but also the topic knowledge that is required in communicative contexts (Douglas, 2013) since communication in the real world often involves content exchange (Basturkmen and Elder, 2004). On the one hand, the evaluation of both knowledge types in a test has the potential to better represent the target language domain and thereby improve test validity. On the other hand, however, it may increase the difficulty of rating and score interpretation, thus having a negative impact on test reliability. It is probably because of this tension, the issue of whether and how linguistic and content knowledge should be assessed in language tests, that CLT test validity and reliability has attracted some attention in the field of English for specific research (Johns, 2012). Yet despite some research having been conducted, no conclusions have yet been reached. These illustrations demonstrate that maintaining a balance between face validity and reliability in CLT remains a challenging issue.

## Directions for future research

Based on the above descriptions of CLT features, approaches, and challenges, this section proposes some potential directions for future research.

- *Improve theoretical models of CLA*. Existing CLA models can be improved in two ways. In terms of content, they can be complemented with theories of L2 learning and development (Purpura, 2008) and incorporate new construct components brought about by the technological revolution (Coiro et al., 2008), such as digital literacies (Gillen and Barton, 2010). In terms of practicality, efforts should be made to simplify complex CLA models into practical step-by-step guidelines so that the constructs of these models can be better operationalized (Harding, 2014).
- *Investigate new tasks that reflect modern digital communication*. Different forms of digital communication, whether they be written or verbal, are widely used in today's technologically advanced world. Language assessment needs to account for these changes that have been brought on by technology (e.g., Teasdale and Leung, 2000; Godwin-Jones, 2008).
- *Explore test takers' adaptability*. Since real-world communication often involves diverse and unfamiliar language varieties (Harding and McNamara, 2017; Harding, 2018), research should investigate test takers' ability “to deal with different varieties of English, to use and understand appropriate pragmatics, to cope with the fluid communication practices of digital environments, and to notice and adapt to the formulaic linguistic patterns associated with different domains of language use” (Harding, 2014, p. 194).
- *Utilize both independent and integrated tasks*. According to the principles of authenticity and interactiveness (Bachman, 1990), integrated tasks are preferred in CLT since they can demonstrate test takers' competence in using multiple skills for effective communication (Cumming, 2013; Plakans et al., 2019; Rukthong and Brunfaut, 2020). Independent tasks, on the other hand, provide more precise information regarding test takers' various language skills, which is beneficial for both teaching and learning. Further research should be conducted to explore how the combination of the two task types can elicit information that is more useful for assessment and pedagogical purposes (Cumming et al., 2005; Plakans, 2010).

## Conclusion

Even though CLT is not new and has become a widely accepted approach for language assessment, test developers still find it challenging to apply CLT models to their test designs (Weir, 2005) due to several unresolved issues. Additionally, CLT is an approach that reflects real communicative activities. Given the rapid advancement of technology, real-world communication has evolved significantly since CLT was first proposed and is still changing. Therefore, our understanding of CLT must keep up with these ongoing changes. In other words, we should not let the discussions of CLT die (Harding, 2014). Motivated by this, this review has gone beyond prior CLT conversations (e.g., Bachman, 1990; McNamara, 1996), and described the dominant CLT approaches and unresolved challenges based on today's communicative practices. It not only provides readers with up-to-date information on communicative competence but also offers guidance for the future development of language tests. We encourage that dynamic, ongoing reviews of CLT be conducted after accounting for the developments of modern real-world communication.

## Author contributions

LL: conceptualization and writing. SY: writing, revising, and editing. JY: revising and visualization. All authors contributed to the article and approved the submitted version.
